# Creating symptom-based criteria for diagnostic testing: a case study based on a multivariate analysis of data collected during the first wave of the COVID-19 pandemic in New Zealand

**DOI:** 10.1186/s12879-021-06810-4

**Published:** 2021-10-30

**Authors:** Nigel French, Geoff Jones, Cord Heuer, Virginia Hope, Sarah Jefferies, Petra Muellner, Andrea McNeill, Stephen Haslett, Patricia Priest

**Affiliations:** 1 Infectious Disease Research Centre, Massey University, Palmerston North, New Zealand; 2 School of Fundamental Science, Massey University, PO Box 11222, Palmerston North, New Zealand; 3 Institute of Environmental Sciences and Research Ltd, Porirua, New Zealand; 4 Epi-Interactive Ltd, Wellington, New Zealand; 5 Department of Preventive & Social Medicine, University of Otago, Dunedin, New Zealand

**Keywords:** COVID-19, Triaging, Symptoms, Epidemiology, Machine learning

## Abstract

**Background:**

Diagnostic testing using PCR is a fundamental component of COVID-19 pandemic control. Criteria for determining who should be tested by PCR vary between countries, and ultimately depend on resource constraints and public health objectives. Decisions are often based on sets of symptoms in individuals presenting to health services, as well as demographic variables, such as age, and travel history. The objective of this study was to determine the sensitivity and specificity of sets of symptoms used for triaging individuals for confirmatory testing, with the aim of optimising public health decision making under different scenarios.

**Methods:**

Data from the first wave of COVID-19 in New Zealand were analysed; comprising 1153 PCR-confirmed and 4750 symptomatic PCR negative individuals. Data were analysed using Multiple Correspondence Analysis (MCA), automated search algorithms, Bayesian Latent Class Analysis, Decision Tree Analysis and Random Forest (RF) machine learning.

**Results:**

Clinical criteria used to guide who should be tested by PCR were based on a set of mostly respiratory symptoms: a new or worsening cough, sore throat, shortness of breath, coryza, anosmia, with or without fever. This set has relatively high sensitivity (> 90%) but low specificity (< 10%), using PCR as a quasi-gold standard. In contrast, a group of mostly non-respiratory symptoms, including weakness, muscle pain, joint pain, headache, anosmia and ageusia, explained more variance in the MCA and were associated with higher specificity, at the cost of reduced sensitivity. Using RF models, the incorporation of 15 common symptoms, age, sex and prioritised ethnicity provided algorithms that were both sensitive and specific (> 85% for both) for predicting PCR outcomes.

**Conclusions:**

If predominantly respiratory symptoms are used for test-triaging,  a large proportion of the individuals being tested may not have COVID-19. This could overwhelm testing capacity and hinder attempts to trace and eliminate infection. Specificity can be increased using alternative rules based on sets of symptoms informed by multivariate analysis and automated search algorithms, albeit at the cost of sensitivity. Both sensitivity and specificity can be improved through machine learning algorithms, incorporating symptom and demographic data, and hence may provide an alternative approach to test-triaging that can be optimised according to prevailing conditions.

**Supplementary Information:**

The online version contains supplementary material available at 10.1186/s12879-021-06810-4.

## Background

PCR testing to identify SARS-CoV-2 cases is a fundamental pillar of pandemic control. The decision on which set of symptoms to use for determining who should be tested by PCR will ultimately depend on resource constraints and public health and disease control objectives in the country and, where possible, aim to minimise test error rates. Therefore, the criteria for PCR test eligibility has varied by time and between countries. For example, at the time of writing, for a person in the community not being investigated as a contact of a known case, in the UK it is necessary to have a fever, a new, continuous cough and loss of sense of taste or smell to be eligible for a government-funded PCR test [1], in Australia anyone with “cold or flu like symptoms, such as a cough, fever, sore throat, shortness of breath or runny nose, even if these are mild” are advised to “get tested for COVID-19 as soon as possible” [2]. In New Zealand anyone with symptoms of COVID-19, listed as a new or worsening cough, fever (at least 38˚C), shortness of breath, a sore throat, sneezing and runny nose, and temporary loss of smell, are advised to contact their doctor or a national health phone line, who will then advise them whether they need testing.

The choice of symptoms that are required to meet the clinical criteria will directly affect the number of individuals put forward for PCR testing and the ability to detect cases in the community. If the chosen combination of symptoms lacks sensitivity, many true cases are likely to be missed, in addition to asymptomatically infected individuals not being captured through screening or contact tracing. If the combination of symptoms lacks specificity, then a large number of individuals will be tested unnecessarily. This has implications for preventing community transmission (lack of sensitivity) and for resourcing (lack of specificity). Therefore, any clinical criteria defined for testing based on symptoms need to balance the need to detect disease to prevent transmission, against the risk of overwhelming the testing and public health services with individuals that are not COVID-19 cases.

To date in New Zealand, an ‘elimination strategy’ [3] has being followed, with an emphasis on maximising the sensitivity of COVID-19 surveillance so that chains of transmission are detected and controlled as early as possible. However, in contexts where case numbers are high and resources are constrained and under pressure, it may be acceptable to miss some cases if it protects resources from being depleted by unnecessary testing. Such contexts may include the implementation of a mitigation approach, or when countries like New Zealand or Australia re-open their borders after mass population COVID-19 vaccination. A scenario in which the importation of new strains of seasonal influenza alongside vaccine sensitive and potentially resistant strains of SARS-CoV-2, increasing the potential of widespread acute respiratory illnesses, may also benefit from an approach that favours high specificity at the cost of a reduced sensitivity.

Other studies have described the use of self-reported symptoms to develop and train models that can identify infection, with the aim of identifying cases early in the course of infection, enabling prompt self-isolation and testing [4–6]. These include the use of large datasets of self-reported symptoms and PCR test results, collected via mobile phone applications, and an array of modelling methods including logistic regression [4, 5] and a hierarchical Gaussian process model [6] and machine learning models [7, 8]. Self-reported symptoms have also been used to identify changes in the symptomatology and disease profiles associated with vaccination [9] and the introduction of new variants of concern [10]. Accurately characterizing the relationship between specific symptoms or clusters of symptoms and the presence or absence of a positive test for COVID-19 may contribute to the formulation and enhancement of case definitions for both acute and ‘long’ COVID-19, and to criteria for prioritising access to testing [11]. Case definitions that are sensitive and specific enhance early case identification and initiation of case and outbreak response.

The aim of this study was to provide a quantitative approach to creating symptom-based criteria for testing using data collected at the time of presentation to health services. We use detailed data collected on New Zealanders tested in the community during the ‘first wave’ of COVID-19 [12] to describe the patterns of clinical symptoms from February to May 2020, and to assess the performance of different combinations of symptoms in terms of their sensitivity and specificity, to inform their potential utility in triaging individuals for PCR testing. We explain how the quantitative techniques that can be used to achieve this could be applied to determine real-time criteria for PCR testing for COVID-19 or other respiratory pathogens in different disease control scenarios.

## Methods

### First wave of COVID-19 in New Zealand

The study period is from the 26^th^ February 2020 to the 9^th^ June 2020, during which time New Zealand recorded 1153 confirmed and 350 probable cases of COVID-19 [12]. Diagnosis of confirmed cases was by real time PCR using WHO recommended primers and probes targeting the *E* and *N* genes [13]. During this first wave of COVID-19 in New Zealand, the decision to test individuals presenting to health services was based on a health practitioner-led risk assessment in the context of the Ministry of Health’s COVID-19 case definitions. The clinical criteria included: “any acute respiratory infection with at least one of the following symptoms: new or worsening cough, sore throat, shortness of breath, coryza, anosmia, with or without fever”. Significant changes to the clinical features in the case definition included: the removal of fever as a requirement on 14 March 2020, and the addition of anosmia or coryza on 01 April 2020.

### Data

All suspect cases of COVID-19 who presented to health services during the study period and were notified to Public Health officials via EpiSurv, the national notifiable disease database, were considered in this analysis (N = 7549). Of these individuals, 7036 were tested by PCR and reported as either positive or negative for SARS-CoV-2. Those with a confirmatory PCR test were classified as confirmed cases. Probable cases were close contacts of confirmed cases with compatible clinical illness where PCR testing results were negative or inconclusive. This analysis includes only the 6224 cases who were classified as symptomatic, and of these 1125 were classified as ‘confirmed’ cases, 349 were classified as ‘probable’ cases and 4750 were classified as ‘not a case’. Patient symptom data were collected at notification along with diagnostic, demographic, outcome and risk factor details using a standardised case report form on EpiSurv,^1^ with most symptoms included as specific yes/no fields. ‘Loss of sense of smell’ (i.e. anosmia) was added as a specific field in April, but was reported under ‘other symptoms’ prior to this. Ageusia was only reported under ‘other symptoms’.

### Variables

Variables included the presence or absence of symptoms and epidemiological variables, including age, sex and prioritised ethnicity. Age and sex were determined from the standardised case report form, and prioritised ethnicity was self-determined and obtained by linkage to the national patient demographics dataset (National Health Index, NHI) and grouped in order of prioritization to Māori, Pacific, Asian then European and Other [14  Ethnicity from the NHI should be self-identified according to health sector protocols, but may be by proxy or assigned [14].

Symptom variables considered in this analysis (and their symbols) are those that occurred in at least 100 individuals: Ageusia (loss or reduction of sense of taste) (**G)**, Anosmia (loss of sense of smell) (**A)**, Coryza (runny nose) (**Z)**, Cough (**C)**, Diarrhoea (**D)**, Fever (**F)**, Headache (**H)**, Nausea/Vomiting (**V),** Pain:Abdomen (**P**), Pain:Chest (**S)**, Pain:Joint (**J),** Pain:Muscle (**M)**, Shortness of Breath (**B)**, Sore Throat (**T)**, General Weakness (**W)**. All symptoms were specific fields in the standard case report form (https://surv.esr.cri.nz/episurv/CaseReportForms/Coronavirus_Sep2020.pdf) with the exception of ageusia which was noted in the free text ‘other symptom’ field.

### Analytical methods

A group of methods was used to explore the datasets with increasing levels of complexity, starting with a simple visualisation of frequency distributions, extending to multivariate analyses and machine learning models.

#### Frequency distributions using UpSet plots

The frequencies of different symptoms and symptom combinations for those classified as ‘Not a case’, ‘Confirmed case’ and ‘Probable case’ were displayed as ‘UpSet’ plots using the R package ComplexHeatmap (https://github.com/jokergoo/ComplexHeatmap).

#### Multiple correspondence analysis

Patterns of symptoms were explored using Multiple Correspondence Analysis (MCA) using the R packages FactoMineR and factoextra [15]. MCA is a factor analysis related to correspondence analysis (CA) and principal component analysis (PCA) [15]. It calculates the chi-square distance between variables from an indicator matrix of: individuals × variables. Variables are factors or dummy variables with value 1/0 (present/absent). Here the matrix columns were the active variables consisting of the 15 most common symptoms, and supplementary variables (status, ethnicity, age group and sex). The calculated distances can be used to represent each variable as a point in space, and this is then mapped into the two dimensions that capture the most variation. The further away from the origin, the greater the contribution to the overall variance; and the closer variable categories are located in the graph, the more likely they are to occur in the same individual [15].

Supplementary variables are not used to calculate the pairwise distances between individuals in the MCA but their coordinates are predicted and plotted on the MCA plot.

The supplementary variables were considered as follows:

*Status: “Not a case”, “Confirmed case” and “Probable case”*

*Prioritised Ethnicity: “Māori”, “Pacific Peoples”, “Asian”, “Middle Eastern/Latin American/African”, “European or Other”, “Unknown”.*

*Age Group: “ < 1”, “1–4”, “5–9”, “10–14”, “15–19”, “20–29”, “30–39”, “40–49”, “50–59”, “60–69”, “70 + ”.*

*Sex: “Male”, “Female”, “Unknown”*

*Month of year: “2”, “3”, “4”, “5” and “6”*

Informed by the MCA, groups of variables linked by Boolean ‘AND” and ‘OR’ statements were assessed against ‘status’ as a quasi-gold standard, after omitting probable cases, to determine the sensitivity and specificity of each set of symptoms. This was augmented by the application of an automated search algorithm (see Additional file 1: Additional material).

#### Decision tree analysis and machine learning/random forests

Decision tree analysis (DTA) partitions a dataset recursively using binary splits based on the predictor variables, at each stage seeking to maximize the ‘purity’ of the components of the partition in terms of the target variable [16]. Here the target was the Status variable, restricted to “Not a case” or “Confirmed case”, and the predictors were the symptoms and supplementary variables used in the MCA analysis. The mean decrease in ‘impurity’ (using the GINI index) is an evaluation of how important removal of a particular variable is on the purity of nodes, where high purity is where each node contains predominantly one outcome (i.e. ‘Confirmed case’ or ‘Not a case’). The maximum purity would be a situation where each node contains only one status outcome (see Additional file 1: Additional material for the results of a simple decision tree analysis).

One of the limitations of simple decision tree analysis is that, unless the dataset is very large, the tree topology can vary markedly, depending on the random selection of the training and test subsets of the full data. An extension of this method is the machine learning method using ‘Random Forests’. This method generates a large number of decision trees, each constructed using a different subset of the full dataset for training. The subsets are selected by sampling at random and with replacement from the full data set. These multiple decision trees (i.e. a forest of trees) are used to determine a classification consensus. One of the outputs of random forests includes a determination of the importance of each variable in terms of its impact on accuracy and ‘impurity’ (using the GINI index). The mean decrease in accuracy for a given variable is an estimate of the loss of prediction performance if that variable is removed from the training dataset. Random forest analysis was performed using the R packages ‘randomForest’ and ‘caret’ [17, 18]. For the random forest analysis both symptoms and supplementary variables were considered. Each model randomly sampled multiple variables as candidates for each split, and grew 500 trees, resampled using tenfold cross validation. The number of variables sampled was determined by an optimisation procedure using Cohen’s Kappa as the evaluation metric.

Both the decision tree analysis and the random forest analysis randomly selected 60% of the 4750 symptomatic ‘not cases’ and 1125 ‘cases’ for training, and 40% for testing. Given the ‘unbalanced’ nature of the dataset a number of approaches were used to optimise the performance of the models in terms of their ability to predict confirmed cases (i.e. sensitivity) and individuals that were not a case (i.e. specificity). These included sampling from the two groups, for example up-sampling the minority class “Confirmed case”, or down-sampling of the majority class “Not a case”, and the use of other similar methods such as the ‘rose’ and ‘smote’ algorithms [19] and varying prediction cut-off values, informed by Receiver Operator Curves (ROCs), using the R package pROC [20].

#### Other analytical techniques

Methods and results of other analyses are included in the Additional file 1: Additional material – an automated exploration of the sensitivity and specificity of symptom combinations using ‘status’ as a quasi-gold standard; Bayesian Latent Class Analysis (BLCA) [21]; and single decision tree analysis – are shown in the Additional file 1: Additional material.

Further details of the implementation of the statistical methods, including code, are available from the authors on request.

## Results

### Frequency distributions using UpSet plots

**Fig. 1:**
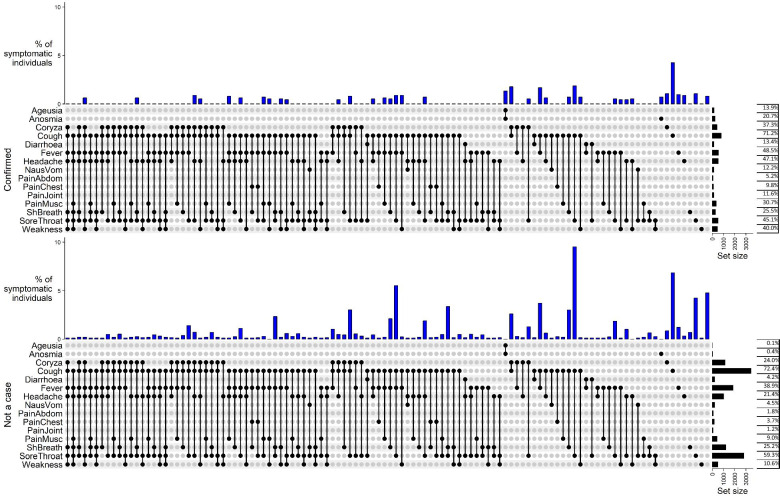
Distribution of the combination of 15 symptoms reported by confirmed and ‘not a case’ individuals displayed using an ‘Upset’ plot. Only symptoms reported in > 100 people and combinations that occurred in 5 or more individuals are included. The total number of PCR confirmed cases (top plot) was 1125 and the total number of non-cases (bottom plot) was 4750. A plot including ‘probable’ cases is provided in Additional file 1: Fig. S1

Frequencies of symptoms for ‘Confirmed’ (N = 1125) and ‘Not a case’ (N = 4750) individuals are shown in Fig. 1. The two most common symptoms were cough (71.2%) and fever (48.5%) for confirmed cases and cough (72.4% and sore throat (59.3%) for non-cases. The most common combinations of symptoms in confirmed cases were cough with sore throat (1.9%), cough with coryza (1.8%) and cough with fever (1.7%), whereas in non-cases the most common combinations were cough with sore throat (9.5%), and the combination of cough, sore throat and fever (6.9%). The combination of ageusia and anosmia was relatively common for cases, but uncommon in ‘not a case’ individuals. Additional file 1: Fig. S1 shows the distributions for all outcomes including 349 ‘probable cases’.

### Multiple correspondence analysis

**Fig. 2:**
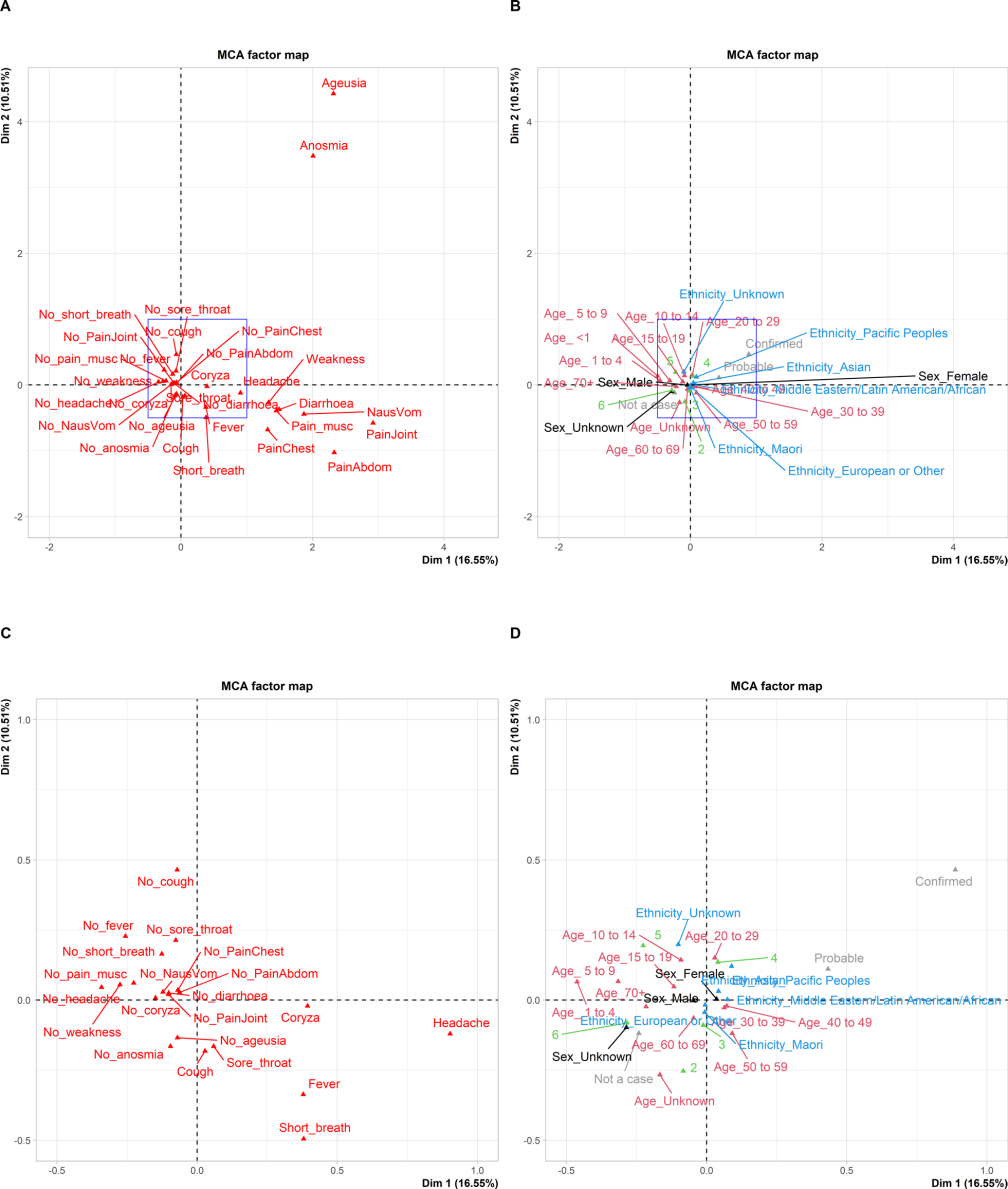
**A**, **B** Multiple Correspondence Analysis of 1125 cases, 349 probable cases and 4750 non-cases presenting for SARS-CoV-2 PCR testing during the first wave of COVID-19 in New Zealand, showing the correlation between symptom variables **A** and supplementary variables **B** with the first two dimensions, which accounted for ~ 30% of the variation. Supplementary variables were age category (magenta), priority ethnicity (blue), sex (black), disease status (grey) and month of year (green). The blue box is expanded in (**C**, **D**). **C**, **D** Multiple Correspondence Analysis showing the correlation between symptom variables **C** and supplementary variables **D** with the first two dimensions, with the axes cropped as indicated by the blue boxes in (**A**, **B**)

Figure 2A–D shows the non-respiratory symptoms (ageusia, anosmia, diarrhoea, headache, nausea/vomiting, abdominal pain, joint pain, muscle pain, and general weakness) were generally more discriminatory than the respiratory symptoms (cough, sore throat and coryza) and closer to the confirmed cases level (i.e. they were further to the right of the null value in Dimension 1 and closer to the status level “confirmed”). For this analysis 1125 cases, 349 probable cases and 4750 non-cases were included.

Figure 2A and C show the correlation between the symptom variable categories and the first two principal dimensions, which captured ~ 30% of the variation between individuals. This is greater than the approximately 13% of variation (2/15) expected if the variables were all uncorrelated, suggesting these two components each represent an important relationship connecting the variables considered. Variables such as joint pain, abdominal pain, nausea and vomiting were most strongly associated with the first dimension (which accounted for ~ 16.5% of the variation), whereas anosmia and ageusia were strongly associated with variation in both the first and second dimensions. In Fig. 2B and D the supplementary variables related to ethnicity, age group and sex were located close to the origin, indicating that these variables were not strongly correlated with individual symptoms.

**Fig. 3:**
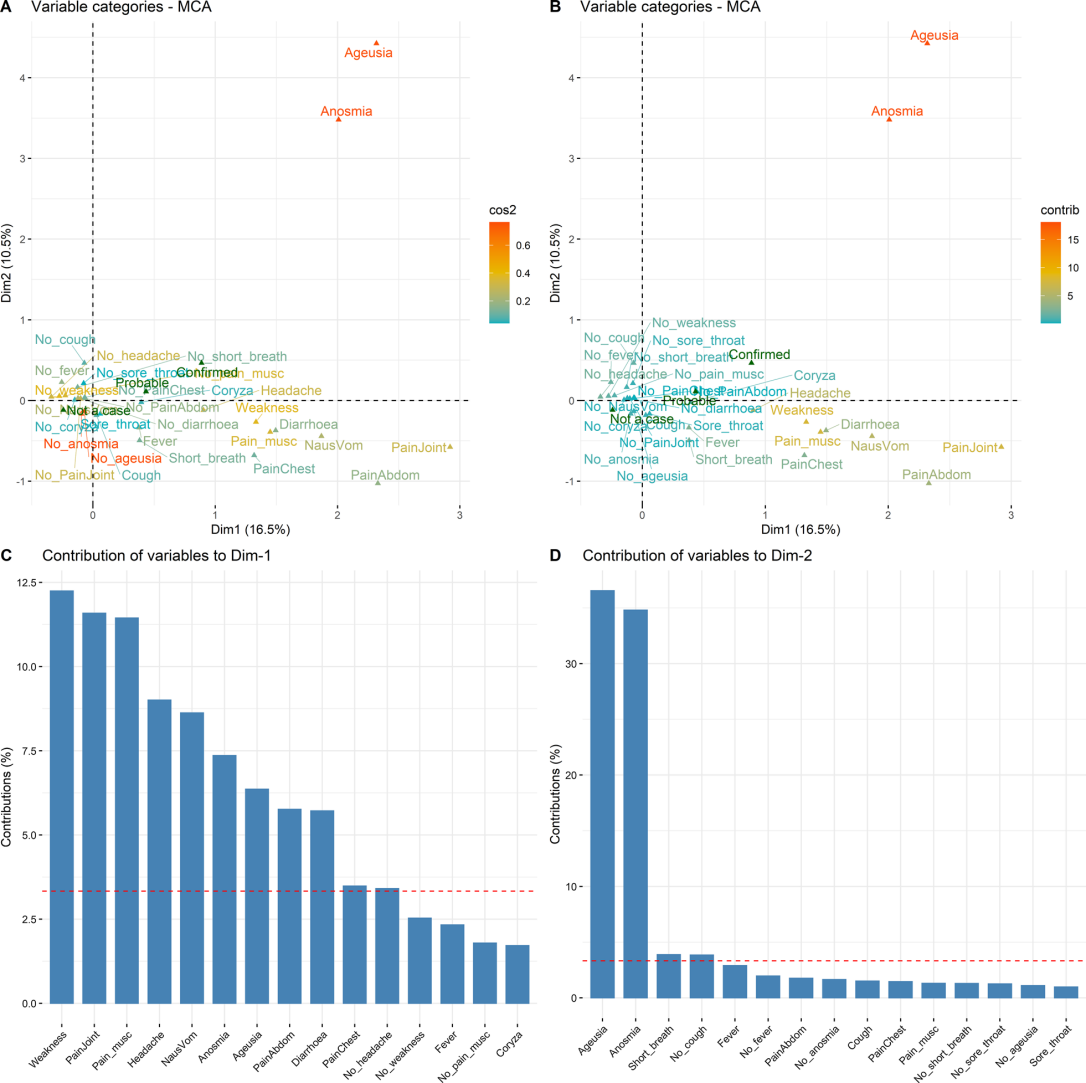
Multiple Correspondence Analysis plots of 1125 cases, 349 probable cases and 4750 non-cases, showing relationships between symptom variables and disease status in individuals presenting for SARS-CoV-2 PCR testing during the first wave of COVID-19 in New Zealand. **A** is coloured according to the quality of representation of each symptom variable category (e.g. Cough and No-cough combined), which is the total of the squared cosine (cos2) values measuring the degree of association between variable categories and each axis, whereas **B** is coloured according to the contribution of each level within variable categories to the definition of the two dimensions (expressed as a %). **C**, **D** show the contribution of each of the 15 most important variables to the variation in dimensions 1 and 2. The red dashed line is the expected average value, assuming all contributions were uniform (i.e. the expected value under the null)

Figure 3 further explores the contribution of each variable to the overall variation in symptoms between individuals, including only disease ‘Status’ as a supplementary variable. Figure 3A is coloured according to the quality of representation of each symptom variable category (e.g. Cough and No-cough combined) whereas Fig. 3B is coloured according to the contribution of each level within variable categories to the definition of the two dimensions. Note that the variable levels anosmia and ageusia are red, indicating a high contribution to the definition of the dimensions, whereas the levels no anosmia and no ageusia are blue, indicating low contribution. Figures 3C, D show the contribution of each variable level to the variation in dimensions 1 and 2, with the dashed red lines indicating what would be expected under the null hypothesis. The locations of all individuals in the first two dimensions, coloured by PCR and symptom variables is shown in Additional file 1: Fig. S2.

Further, symptoms (present or absent) which occur more frequently within the same individual tend to group together on the plot, whereas symptoms that are on opposite sides of the plot origin (i.e. opposed quadrants) tended not to occur in the same individual. For example, diarrhoea, chest pain, muscle pain and general weakness tended to occur together, whereas fever and anosmia were less likely to occur together. Categories that are most discriminatory (i.e. explain relatively more of the variation in the data) tend to be furthest away from the origin (i.e. further from where the two dimensions intersect). Anosmia, ageusia and joint pain are good examples of symptoms that were very discriminatory, and that often occurred in the same individual.

The MCA analysis, in particular the position of the symptoms per se and their location relative to the ‘Status’ variables, was used to propose and compare combinations of symptoms in terms of their ability to predict confirmed cases and individuals that were not cases. This is analogous to determining the sensitivity and specificity of clinical signs using the ‘status’ variable (‘confirmed cases’ or ‘not a case’) as a quasi-gold standard. Note, in this analysis we just consider individuals presenting with clinical symptoms (the BLCA analysis described in the Additional file 1: Additional material considers both symptomatic and asymptomatic individuals and assumes there is no gold standard).

We can use this analysis to identify groups of symptoms and signs that could trigger PCR testing to optimise disease detection under different response scenarios (i.e. different acceptable limits of sensitivity and specificity). Table 1 compares the current case definition ‘Any acute respiratory infection with at least one of the following symptoms: anosmia, coryza, new or worsening cough, shortness of breath, sore throat, with or without fever’ (denoted as A|Z|C|B|T, where | signifies the Boolean term OR)^2^ with two collections of five symptoms identified as discriminatory in the MCA: at least one of the symptoms ‘ageusia, anosmia, nausea/vomiting, abdominal pain, or joint pain’ (G|A|V|P|J) and ‘anosmia, diarrhoea, headache, muscle pain, or general weakness’ (A|D|H|M|W). Note the current case definition is largely based on respiratory symptoms, while the other combinations considered here are based predominantly on non-respiratory symptoms.

**Table.1:** Proportion of cases and not cases with each combination of symptoms

	Symptom combination		
Status	A|Z|C|B|T	A|D|H|M|W	G|A|V|P|J
% confirmed cases with symptoms (N)—i.e. sensitivity	92.2% (1037)	77.2% (868)	39.3% (442)
% not a case without symptoms (N)—i.e. specificity	8.0% (380)	67.4% (3203)	92.9% (4415)
% not a case with symptoms (N)—i.e. false + ves	92.0% (4370)	32.6% (1547)	7.1% (335)
Total number recommended for PCR	5407	2415	777
Number cases not recommended for PCR	88	257	683

Here sensitivity and specificity are calculated using ‘status’ as a quasi-gold standard

Table 1 shows that a respiratory-based designation results in relatively high sensitivity (92%) for COVID-19 PCR positivity. However, it was also a common pattern of symptoms in individuals that were not confirmed cases and hence results in very low specificity (8%). Although the other ‘non-respiratory’ patterns (A|D|H|M|W) and (G|A|V|P|J) occurred in relatively fewer cases (sensitivity = 77% and 39%) they were less commonly observed in individuals that were not cases and therefore had a much higher specificity (specificity = 67% and 93%).

During high incidence scenarios, where health services are overwhelmed, there may be an urgent need to reduce the burden on testing stations by optimising screening using clinical symptoms. This could be achieved by choosing a set of non-respiratory symptoms to triage individuals with enough respiratory symptoms to consider themselves possibly having COVID-19, and hence presenting to health services for possible testing. This would result in many fewer individuals being put forward for testing, with a relatively modest reduction in sensitivity. The first non-respiratory set (A|D|H|M|W) would have resulted in ~ 3,000 fewer PCR tests (5407–2415 in Table 1), but at the cost of missing 257/1125 cases. The second non-respiratory set (G|A|V|P|J) achieved very high specificity (93%), but at the cost of low sensitivity. The decision whether to opt for maximising sensitivity or specificity will depend on the setting and will need to consider the extent of community transmission as well as testing capacity.

### Sensitivity and specificity of multiple symptom sets using ‘status’ as a quasi-gold standard

**Fig. 4:**
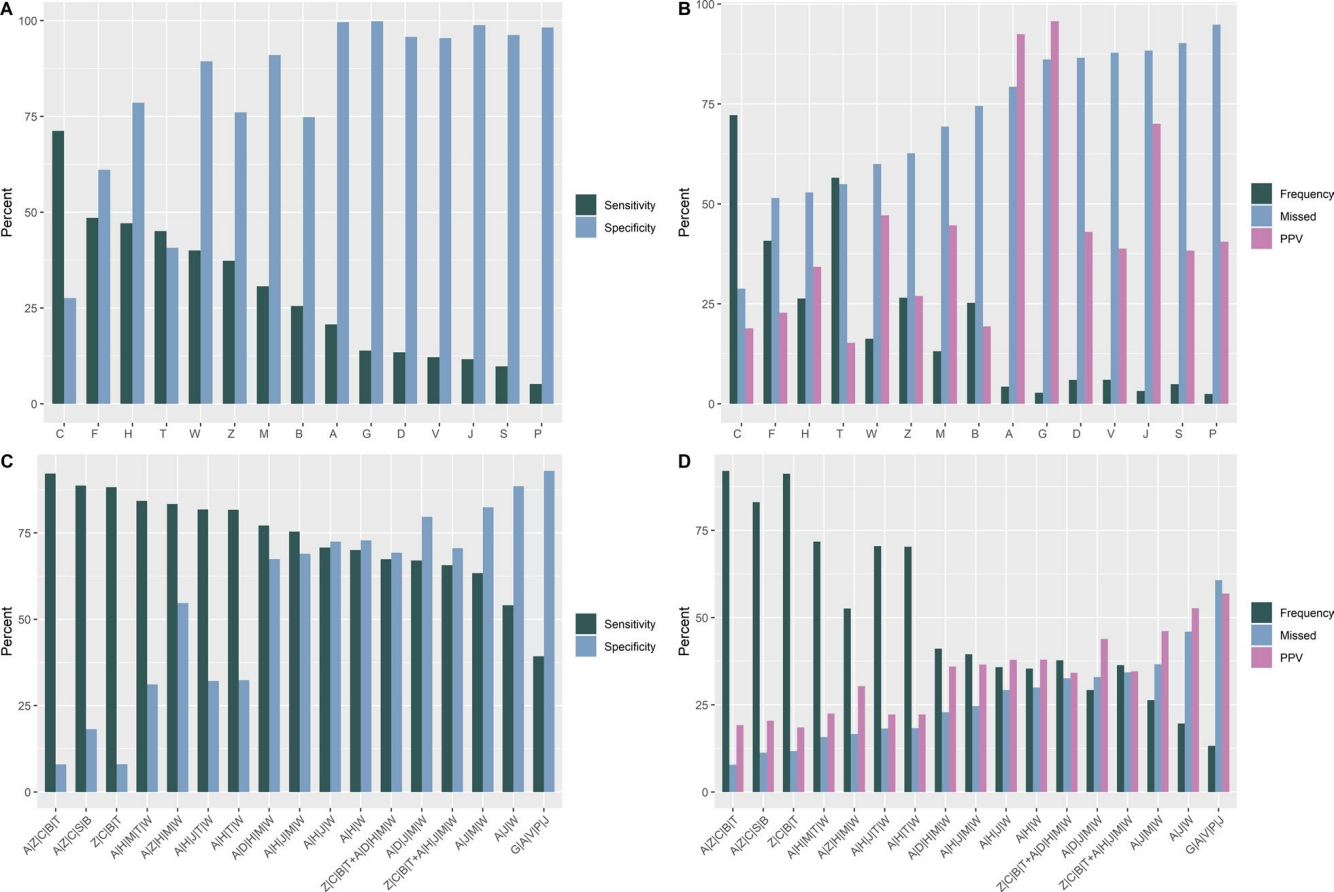
Examples of the characteristics of different individual symptoms and combinations of symptoms linked by ‘OR’ statements in individuals (1125 cases, and 4750 non-cases) presenting for SARS-CoV-2 PCR testing during the first wave of COVID-19 in New Zealand. **A**, **B** show the sensitivity, specificity, positive predictive value and proportion of false negatives (i.e. 1-sensitivity, labelled as ‘missed’) assuming the PCR is a gold standard, and the frequency of individual symptoms, expressed as percentages. **C**, **D** show the same metrics for a selection of combinations of symptoms. The examples ZCTB_AHJMW and ZCTB_ADHMW are rules based on one or more of four respiratory symptoms (coryza, cough, sore throat or shortness of breath, Z|C|T|B) AND one or more of 5 non-respiratory symptoms

We can extend the analysis using the insight provided by the MCA analysis and automate the exploration of various combinations of symptoms, using AND/OR statements. This is described in the Additional file 1: Additional material, where Additional file 1: Fig. S3A, B show scatterplots of different combinations of symptoms linked by Boolean ‘OR’ statements, according to their sensitivity and false positive rates (i.e. 1-specificity). Figure 4 shows the sensitivity and specificity for individual symptoms (4A) and example groups of symptoms (4C) symptoms using data from 1125 cases and 4750 non-cases. Figure 4B and D indicate the positive predictive values, frequency of symptoms and combinations of symptoms and the percentage of cases that would missed (i.e. the false negatives) for individual symptoms (4B) and combinations of symptoms (4D). The combinations of symptoms that have moderate to high sensitivity and specificity are those identified in Additional file 1: Fig. S3.

A Bayesian Latent Class Analysis assuming no gold standard is described in the Additional file 1: Additional material. Using the BLCA approach the sensitivity and specificity of both symptoms and the PCR assay are estimated (see Additional file 1: Figs. S4 and S5).

### Machine learning and random forest models

**Fig. 5:**
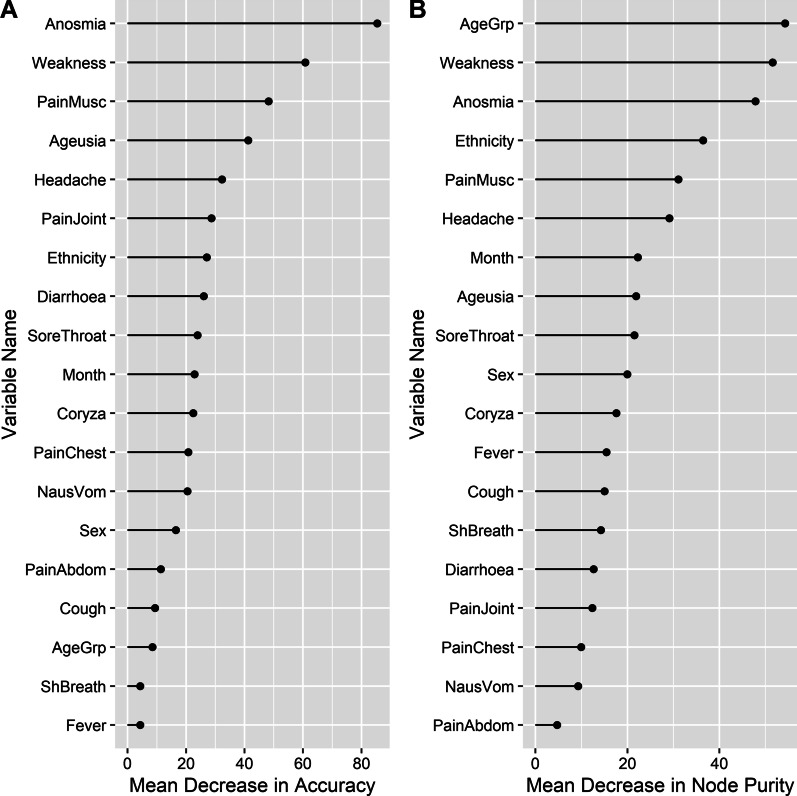
Variable importance analysis using a random forests machine learning algorithm to determine the relative importance of symptom and demographic variables as determinants of the outcome of SARS-CoV-2 PCR testing, using 1125 cases and 4750 non-cases presenting for testing during the first wave of COVID-19 in New Zealand. The mean decrease in accuracy measures the importance of removing each variable on the predictive accuracy of the model, whereas the mean decrease in purity measures the importance of removing each variable on node ‘purity’ (see Methods)

An example of a single decision tree is given in Additional file 1: Fig. S6. This was extended using a random forest analysis which confirmed the relative importance of symptom variables identified in the previous analyses, such as anosmia, general weakness, muscle pain and joint pain, in terms of the ability to accurately predict the outcome (i.e. ‘status’). For these analyses 1125 cases, 349 probable cases and 4750 non-cases were included. Some of the demographic variables, most notably age group and ethnicity, were relatively important in determining node purity (Fig. 5). Therefore these variables, as well as sex, were included in subsequent analyses as they are easily recorded and are likely to improve the sensitivity and specificity of random forest models developed to support decision making.

**Fig. 6:**
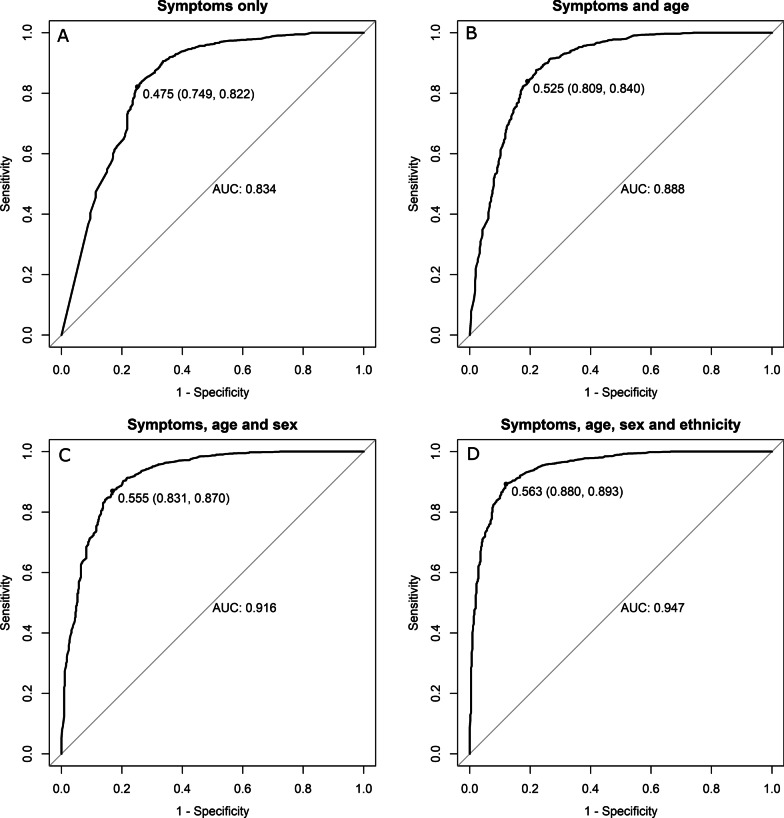
Receiver Operator Curves (ROCs) showing the relationship between sensitivity and specificity for different cut-off values used to determine a recommendation to test for SARS-CoV-2 by PCR based on predicted probabilities of being a case from machine learning / random forest models. The dataset comprised 1125 cases and 4750 non-cases presenting for testing during the first wave of COVID-19 in New Zealand. For example, **B** shows the relationship between sensitivity and specificity for a random forest model including 15 symptom variables and age group. The optimal cut-off, 0.53 (i.e. the point closest to the top left corner), maximises the sensitivity and specificity of the decision to test by PCR, comparing the model predictions produced by the training set with the actual status (confirmed or not a case) in the test set. In this example the sensitivity is 84% and the specificity of 81%. The area under the curve (AUC) is also provided and illustrates how the predictions improve as more covariates are added from (**A**–**D**)

Random forest models were extended by including more demographic covariates. These were used to develop algorithms on the training datasets that could be used to predict the outcome ‘status’ on the test datasets. The aim here was to use the predicted probability of being a confirmed case to inform a rule for selecting individuals for PCR testing. The sensitivity and specificity of the rule depends on the cut-off value for the predicted probabilities; the optimal value being the point nearest to the top corner of the Receiver Operator Curve (ROC, a plot of sensitivity against false positive rate or 1-specificity). The effect of varying the cut-off value and the inclusion of additional covariate is illustrated in Fig. 6.

The inclusion of more predictor variables improves the performance of the model, as determined by the area under the curve (AUC) moving from Fig. 6A–D. A model that includes the 15 symptoms, plus age and sex achieved 87% sensitivity and 83% specificity at the optimal cut-off value of 0.56. The addition of prioritised ethnicity increased the sensitivity to 89% and specificity to 88% at a similar optimal cut-off value.

## Discussion

The symptom analyses presented here were performed on observational data routinely collected from patients presenting with acute respiratory infections during the first wave of COVID-19 in New Zealand. Similar analyses using machine learning algorithms for test prioritisation based on symptoms have been conducted using data from other countries including Israel and Brazil [22–24]. Given New Zealand’s current elimination strategy [3], high sensitivity in the targeting of testing to clinically compatible COVID-19 presentations is favoured over specificity during this initial phase of the pandemic, but this balance is likely to change with the implementation of the vaccination programme, opening of borders and increasing prevalence of seasonal acute respiratory illness. The nature of the analysis presented provides an opportunity for results to be continuously updated as new data informs the model outputs and could be adapted to consider data from other diagnostic assays with different test characteristics, such as PCR testing of saliva. Hence results could inform future testing strategies and, if processed in real-time, could account for changes in symptoms associated with newly emerged variants. Further, this work could inform the development of targeted testing protocols, such as repeated testing of employees working at international borders; including those who present with clinical illness and are initially test negative. In the setting of endemic disease and mitigation strategies which aim to prevent severe disease and health system overwhelm, improving the specificity of the symptoms which require self-isolation or quarantine and subsequent testing may be useful. This could minimise the potential negative consequences of the widespread use of quarantine and testing, not only on finite resources in the COVID-19 response, but on personal loss of income, for example, or hesitancy to get tested among frequently tested or socially disadvantaged groups.

Although a variety of approaches have been used in this analysis of the dataset of individuals reporting COVID-19 symptoms in New Zealand, the inferences are broadly consistent. The data support the following conclusions:

- Clinical criteria used to guide who should be tested by PCR based on one or more of a set of mostly respiratory symptoms (a new or worsening cough, sore throat, shortness of breath, coryza, anosmia, with or without fever, A|Z|C|F|S|B) has high sensitivity (> 90%) but very low specificity (< 10%). Use of the respiratory symptom criteria alone will therefore result in a large proportion of individuals being tested that do not have COVID-19. Depending on outbreak size, this could overwhelm community testing capacity at sampling/testing stations and laboratories and negatively affect the country’s ability to successfully trace and eliminate.
- Alternative ‘intuitive’ rules combining symptoms with AND/OR statements can increase specificity, but this is inevitably at the cost of sensitivity, with the inherent risk of missing true cases. It is noted that infected individuals without symptoms would be missed completely if triaging were exclusively based on clinical signs [25], and some symptomatic cases, particularly those with mild symptoms, may not present or be reported [26, 27]. Thus, high sensitivity of detection of all infected individuals, including asymptomatic individuals, would not be achieved under any scenario of community transmission, including community transmission under an elimination scenario.
- Marked improvements in both the sensitivity and specificity of rules based on machine learning algorithms can be achieved using decision tree and random forest methods that include both symptoms and epidemiological/demographic variables, such as age and sex. Predictions based on these algorithms can achieve sensitivities and specificities that are both greater than 80% and could be incorporated into easy to access decision support tools [28], such as mobile phone applications, made available to medical practitioners for triaging individuals for PCR testing. These applications are generic and could be adapted as outbreak management tools for multiple diseases.

A study analysing the epidemiology of COVID-19 in the United Kingdom used information from approximately one million individuals with a valid swab test, of which only 11.1% recorded symptoms. Seven symptoms were reliably associated with a PCR positive swab: anosmia, ageusia, fever, new persistent cough, chills, appetite loss and muscle aches [4, 5]. Many of these were similar to those symptoms identified in the New Zealand dataset. To date there have been relatively few other reported studies of COVID-19 symptoms, and even fewer reporting their use to inform public health actions or clinical decision making [4–7, 9, 10, 29, 30]. A study using data from the UK Covid Symptom Smartphone Application used a similar approach to the New Zealand study, combining symptom data with demographic information to predict COVID-19 PCR test positivity [6]. Models were trained using data collected at several time points and showed a similar level of prediction of test positivity to the New Zealand random forest model (AUC ~ 0.8). An example of an application for clinical decision making is a study that analysed six distinct symptom presentations with the aim of identifying individuals needing respiratory support. This study, also using data from the UK Covid Symptom Smartphone Application, employed unsupervised time series clustering [8].

The symptoms anosmia and ageusia, although relatively rare, were shown to be highly discriminatory and specific in this study, and this is consistent with other reports [4, 7, 31]. It should be recognised, however, that a diminished sense of smell is relatively common, especially in older people, and may limit recommendations for screening based on this symptom. Concerns have been raised about the use of this symptom as a screen for limiting access to high risk settings as it could introduce a form of discrimination; disadvantaging a proportion of the population beyond the potential benefits to public health [32].

While the application of ‘intuitive’ and more advanced model-based approaches can appear to perform well for predicting COVID-19 status in the symptom dataset provided for this study, it is important to consider other limitations of this approach. These include, but are not limited to, the following:

- There are likely to be major biases in reporting of symptoms. Many individuals were involved in the recording of symptom data and there are likely to be differences in the way questions were asked and interpreted. Recall bias is likely to be important, and the ability to determine certain symptoms in very young children, such as anosmia and ageusia. Similarly, the ‘worried well’ and raised media awareness may have contributed to over-reporting of symptoms in individuals keen to be tested for COVID-19, whereas the lack of inclusion of symptoms such as ageusia in the standard report form (it was only noted under ‘other symptoms’), may have resulted in them being underreported.
- Reporting bias is likely to vary over time. For example, ageusia, anosmia and coryza were not included in the set of standard symptoms in New Zealand until March/April, although they were recorded in free-text fields. We partially addressed this by including ‘calendar month’ as a categorical variable in multivariate analyses; despite the temporal variation in reporting of symptoms, this variable did not emerge as an important predictor in any of the analyses.
- Symptoms at the time of presentation/testing may not be representative of the symptoms an infected or non-infected individual experienced over the time of their infection. For example, some symptoms such as body aches or anosmia may have developed later in the time course of infection. Other similar studies have considered symptoms recorded over multiple time points [6], but the data available for the New Zealand study were collected at a single time point, namely the time the sample was taken.
- In the multivariate analyses all individuals were analysed together regardless of whether they presented as ‘sought healthcare’, ‘contact’, ‘point of entry’ ‘repatriation’, ‘unknown’ or ‘blank’ (the last two making up the majority of individuals). Inclusion of this as a variable in the multivariate analysis was not informative, and subset analyses (e.g. just ‘sought healthcare’) did not reveal any major differences in the conclusions.
- The data available for analysis are a by-product of the monitoring and control measures put in place for COVID-19 in New Zealand. The data are observational and have not been collected via a probability-based sampling scheme. Ideally, data would have been collected for the specific purpose of estimating sensitivities and specificities and would have involved random sampling of the target population and the collection of data on other potential confounding variables. However, even if an *a-priori* sampling design had been intended, there was no clear definition of the target population at the time. As the first wave evolved in New Zealand, selection criteria for most individuals triaged for testing was based on symptoms, and the choice of symptoms then changed over time. Thus, some degree of sampling bias could not be ruled out. To mitigate sampling issues, we used different subgroups in the LCA analysis. The general consistency of the results and conclusions from these different subgroups, while not emphatic, provides useful evidence in support of the choice of symptoms developed in the paper. It suggests that results are robust enough to apply to different populations including other countries and jurisdictions.
- The specificity of algorithms based on symptoms will vary according to the prevalence of other diseases in the population. For example, during periods when respiratory allergies are more common, the specificity of symptoms associated with coryza will reduce, whereas when other viral infections such as influenza or rhinovirus infection are more prevalent symptoms such as fever, headache, joint pain and muscle pain may have lower specificity. Border closures and lockdown occurred in March in New Zealand, ahead of the Southern Hemisphere influenza season, meaning these data were collected during a period of low prevalence of common seasonal respiratory viruses. The effect of varying prevalence of COVID-19-associated symptoms in the populations attributable to other conditions will be considered in a follow-on analysis of symptoms recorded during periods in which other respiratory infections are more prevalent.
- The random forest model performed well at predicting test data from training data, particularly when more demographic variables were included. However, the model with more variables predicts outcomes for much larger arrays of covariate patterns and is less intuitive. For example, a rule to test an individual may indicate different outcomes according to ethnicity, when all other variables, such as symptoms, age and sex, are the same. The age, sex, and ethnicity of cases in NZ were determined by the features of the first wave of the pandemic in New Zealand, associated with large linked clusters of cases and a strong impact of travel-associated cases, and this will affect the generalisability of the models and predictions.
- The addition of prioritised ethnicity to the random forest machine learning algorithm resulted in a marginal improvement in the sensitivity and specificity. However, extreme care would need to be taken when considering the addition of ethnicity as a variable, particularly given the well-described inequalities in health outcomes associated with Māori and Pacific populations in New Zealand [33–35] and the documented misclassification of 21% of Māori as non-Māori [36]. Māori and Pacific populations were relatively underrepresented in the first wave of the pandemic in New Zealand [12] and, although testing rates were comparable with other ethnicities [12], the proportion of tests that were positive was relatively low for Māori (13.4% compared to 18.3 -22.9% for other ethnicities, data not shown). This relatively low test positivity rate resulted in a higher proportion of covariate patterns including Māori ethnicity that were predicted not to be positive by PCR, compared to other ethnicities. If an algorithm informed by test data from the first wave including ethnicity were to be applied for triaging, this could result in Māori being more likely to be excluded from confirmatory testing, despite having the same set of symptoms as other ethnicities. Under a scenario of widespread community transmission, Māori are more likely to be represented among cases, compared to the more travel-associated cases in the first wave. For these reasons, we would not recommend the inclusion of ethnicity in a machine learning-based triaging algorithm and would advise applying similar caution to the inclusion of other demographic variables.

After elimination of the first wave of COVID-19 in New Zealand, the country experienced a series of smaller outbreaks of community transmission, which were eliminated by the application of public health measures, including national and regional lockdowns [37]. In August 2021 New Zealand experienced the start of a large outbreak associated with the Delta variant of SARS-CoV-2. Detailed data on symptoms and testing are currently being gathered in this outbreak, which will be compared with data from the first outbreak to determine if there are changes in patterns of symptoms associated with this variant. Despite anecdotal reports, published studies to date have not identified changes in symptoms associated with different variants [10].

## Conclusions

Using data from the first wave of the COVID-19 pandemic in New Zealand this paper describes how symptom-based criteria for diagnostic testing can be systematically derived, updated and applied to support control of COVID-19. A specific application of this approach could be the development of a tool, based on a machine learning algorithm informed by surveillance data, that guides policy for testing and aids public health decision making for the prevention and control of COVID-19 in the event of sustained community transmission. Updating models as new data become available is likely to improve their performance as a tool for selecting individuals for testing under different scenarios; including a change in the prevalence of other respiratory diseases, the introduction of alternative diagnostic assays, or a change in the priorities of a control programme. This is practically relevant for COVID-19, given the large number of symptoms and symptom combinations associated with the disease. Depending on the pandemic control phase, algorithms using clinical criteria to guide testing might be focussed on avoiding unnecessary testing to protect resources or driven by the need to detect as many cases as possible. The ability to define symptom-based criteria derived from country specific data provides an opportunity for countries to fine tune and adapt their testing strategies to balance the need to detect disease to prevent transmission, against the risk of overwhelming the testing and public health services with individuals that are not COVID-19 cases. Applying such an approach, using a combination of multivariate methods and machine learning, may allow countries to develop and continually update models, using evidence from ongoing surveillance, to support decision making tailored to specific phases of a disease control programme.

## Supplementary Information

**Additional file 1. **Additional material. **Figure S1.** Distribution of the combination of 15 symptoms reported by confirmed, probable and ‘not a case’ individuals. Only symptoms reported in >100 people and combinations that occurred in 5 or more individuals are included. **Figure S2. **Multiple correspondence analysis plots showing the location of each individual in the first two dimensions, coloured by their PCR status (plot A), and symptoms (plots B-L) and 90% prediction ellipses . Plots B and C are symptoms strongly correlated with the second dimension and determine the 3 distinct groups. The upper group are individuals with ageusia and anosmia , the middle group are more likely to be those with anosmia but no ageusia, and the lower group are those without ageusia or anosmia. Plots D-L are symptoms more strongly correlated with the first dimension (positive individuals are more likely to be to the right of all three clusters). Plots K and L are examples of two respiratory symptoms that are not correlated with either dimension. **Figure S3. **Scatterplot of sensitivity and 1-specificity for combinations of symptom variables using ‘status’ as a gold standard. **A** A rule based on combinations of at least one of 5 symptoms. The red dot in the left plot identifies the current case definition based on respiratory symptoms (A|Z|C|B|T). The green and blue dots are sets of largely non-respiratory symptoms used for comparison. **B** A rule based on one or more of respiratory symptoms (coryza, cough, sore throat or shortness of breath, Z|C|B|T) AND one or more of 5 non-respiratory symptoms. The table shows the estimated values for the example sets highlighted in the figure. **Figure S4.** Posterior distributions of the sensitivity (upper row) and specificity (lower row) of both the symptoms (headache OR general weakness OR anosmia OR muscle pain OR joint pain) and the PCR assay, estimated using Bayesian Latent Class Analysis, based on three chains of 20,000 iterations after a burn-in of 5,000. **Figure S5. **Scatterplot of sensitivity and 1-specificity for combinations of symptom variables estimated using Bayesian Latent Class Analysis. **A** A rule based on combinations of at least one of 5 symptoms. The red dot in the left plot identifies the current case definition based on respiratory symptoms (A|Z|C|S|B). The green and blue dots are sets of largely non-respiratory symptoms used for comparison. **B** A rule based on one or more of respiratory symptoms (cough, sore throat, coryza or shortness of breath, Z|C|S|B) AND one or more of 5 non-respiratory symptoms. **Figure S6.** Decision tree built using a machine learning algorithm that minimises the misclassification of cases and non-cases. The thickness of the lines denotes the proportion of the population heading down each branch. Darker shading indicates greater node purity.

## Data Availability

All study data were extracted from the national databases EpiSurv and Éclair, and may be obtained for research purposes by contacting *data-enquiries@health.govt.nz* and following the COVID-19 data request process.

## Footnotes

1 Case report form for EpiSurv. https://surv.esr.cri.nz/episurv/CaseReportForms/Coronavirus_Sep2020.pdf.

2 See methods section for the key to symptom symbols.
