# Supplementary material for: Creating symptom-based criteria for diagnostic testing: a case study based on a multivariate analysis of data collected during the first wave of the COVID-19 pandemic in New Zealand
Source: BMC Infect Dis. 2021 Oct 30;21:1119. doi: 10.1186/s12879-021-06810-4 (PMC8556148; doi:10.1186/s12879-021-06810-4)
Supplement: Supplementary file 1 — Additional file 1. Additional material. Figure S1. Distribution of the combination of 15 symptoms reported by confirmed, probable and ‘not a case’ individuals. Only symptoms reported in >100 people and combinations that occurred in 5 or more individuals are included. Figure S2. Multiple correspondence analysis plots showing the location of each individual in the first two dimensions, coloured by their PCR status (plot A), and symptoms (plots B-L) and 90% prediction ellipses . Plots B and C are symptoms strongly correlated with the second dimension and determine the 3 distinct groups. The upper group are individuals with ageusia and anosmia , the middle group are more likely to be those with anosmia but no ageusia, and the lower group are those without ageusia or anosmia. Plots D-L are symptoms more strongly correlated with the first dimension (positive individuals are more likely to be to the right of all three clusters). Plots K and L are examples of two respiratory symptoms that are not correlated with either dimension. Figure S3. Scatterplot of sensitivity and 1-specificity for combinations of symptom variables using ‘status’ as a gold standard. A A rule based on combinations of at least one of 5 symptoms. The red dot in the left plot identifies the current case definition based on respiratory symptoms (A|Z|C|B|T). The green and blue dots are sets of largely non-respiratory symptoms used for comparison. B A rule based on one or more of respiratory symptoms (coryza, cough, sore throat or shortness of breath, Z|C|B|T) AND one or more of 5 non-respiratory symptoms. The table shows the estimated values for the example sets highlighted in the figure. Figure S4. Posterior distributions of the sensitivity (upper row) and specificity (lower row) of both the symptoms (headache OR general weakness OR anosmia OR muscle pain OR joint pain) and the PCR assay, estimated using Bayesian Latent Class Analysis, based on three chains of 20,000 iterations after a burn-in of 5,000. F [file 12879_2021_6810_MOESM1_ESM.docx]

Additional file 1: MATERIAL

Sensitivity and specificity of multiple symptom sets using ‘status’ as a quasi-gold standard

Using the insight provided by the MCA analysis, the exploration of various combinations of symptoms was automated, using AND/OR statements. Supplementary Figure 3A shows a scatterplot of different combinations of five symptoms linked by ‘OR’ statements, according to their sensitivity and false positive rates (i.e. 1-specificity). While there is some trade-off between sensitivity and specificity, this analysis provides further evidence that combinations of non-respiratory signs provide much higher specificity than the current respiratory set, with a relatively minor loss of sensitivity for some sets. This analysis was further extended to consider intuitive decision algorithms based on the presence of a respiratory symptom used in the current case definition (Z|C|S| B) and one of a set of four or five non-respiratory symptoms (Supplementary Figure 3B). These sets have lower sensitivity and marginally higher specificity compared to the non-respiratory sets.

Sensitivity and specificity of multiple symptom sets using Bayesian Latent Class Analysis

Bayesian latent class analysis [1] was used for simultaneous estimation of the Se/Sp of clinical signs and RT-PCR, without making the assumption that RT-PCR is 100% accurate.

The principle is as follows: if two tests are applied to data from a single population, the outcome can be shown as a 2 × 2 table with test combinations pos/pos, neg/neg, neg/pos and pos/neg. The table has 3 degrees of freedom for estimating 5 parameters (2 × Se, 2 x Sp, 1 × prevalence) – hence there is insufficient information to estimate the parameters. If, however, the tests were applied to an additional second population, there would be two 2 × 2 tables with 6 degrees of freedom for 6 parameters equivalent to 6 equations with 6 unknowns – which can be solved algebraically. The two populations here could be test-patients who were known to have had contact with a COVID-19 case and patients for whom the reason for testing was unknown (blanks or ‘unknown’).

Important assumptions are:

- The accuracy of both tests are identical when used in the selected populations. This is not necessarily plausible in this example. Patients with clinical signs may excrete more virus than asymptomatic people without clinical signs. . Since most tested people in the dataset also had clinical signs and any reason for testing usually included clinical signs, the proportion of patients with clinical signs was similar across the chosen sub-populations. Thus, the assumption was reasonable for this population of individuals.
- The tests are ‘conditionally independent’, i.e. the knowledge of one test result on an infected individual does not predict the result of the second test, and the same holds for non-infected individuals. This assumption is reasonable when the two tests measure different ‘substrates’. In this example, PCR detects RNA on mucosal surfaces and clinical signs develop as a general health response.

The analysis was extended to using three populations: those ‘in-contact’, those who ‘sought healthcare’ and ‘blanks’. Results were compared to evaluate the consistency or lack thereof of the estimated parameters.

Supplementary Figure 4 is an example of a BLCA analysis comparing the symptom set anosmia OR headache OR general weakness OR muscle pain OR joint pain (A|H|W|M|J) with the PCR for COVID-19. In this analysis the sensitivity and specificity of the symptoms are approximately 72-77% and 75-78% respectively, whereas the sensitivity and specificity of the PCR are approximately 68-77% and >99.8% respectively. Supplementary Figure 5 shows a scatterplot of the sensitivity and specificity of multiple combinations of symptoms and the PCR assay estimated using BLCA. The posterior distributions for other combinations highlighted is provided in the appendix.

Decision Tree Analysis – single decision trees

This analysis uses recursive partitioning to optimise the classification of individuals as either ‘confirmed’ or ‘not a case’ according to their clinical symptoms. The symptomatic individuals comprising 1125 confirmed cases and 4750 individuals that were not a case, and the subset of 15 symptoms occurring in more than 100 individuals were used for building trees. The analysis was implemented using the data mining software SAS® Enterprise Miner™ [2], and randomly selected 60% of the each group for training, and 40% for testing. The resulting tree shows, at the terminal nodes, the estimated proportions of cases and non-cases that would result from using the decision rules for triage. For the DTA only the subset of 15 symptoms occurring in more than 100 individuals was used for building trees.

Supplementary Figure 6 is a tree that minimises the misclassification rate (i.e. the proportion misclassified as a case when they were not a case or vice versa). It includes the variables previously identified as optimal in terms of maximising both sensitivity and specificity, but with the addition of ‘sore throat’ as a symptom. However, note that ‘sore throat’ was negatively associated with the outcome; at the final branch in the tree the absence of a sore throat was associated with being a confirmed case. Such an algorithm would result in a similar sensitivity and specificity to the sets identified in the previous analysis (i.e. A|D|H|M|W and A|H|J|M|W) but would be more difficult to implement.

**References for supplementary information**

1. Joseph L, Gyorkos TW, Coupal L: Bayesian estimation of disease prevalence and the parameters of diagnostic tests in the absence of a gold standard. Am J Epidemiol. 1995;141(3):263-272.

2. Matignon R: Data mining using SAS enterprise miner, vol. 638: John Wiley & Sons; 2007.

**Supplementary figure legends**

Additional file 1: **Figure 1.** Distribution of the combination of 15 symptoms reported by confirmed, probable and ‘not a case’ individuals. Only symptoms reported in >100 people and combinations that occurred in 5 or more individuals are included.

Additional file 1: **Figure 2.** Multiple correspondence analysis plots showing the location of each individual in the first two dimensions, coloured by their PCR status (plot A) , and symptoms (plots B-L) and 90% prediction ellipses . Plots B and C are symptoms strongly correlated with the second dimension and determine the 3 distinct groups. The upper group are individuals with ageusia and anosmia , the middle group are more likely to be those with anosmia but no ageusia, and the lower group are those without ageusia or anosmia. Plots D-L are symptoms more strongly correlated with the first dimension (positive individuals are more likely to be to the right of all three clusters). Plots K and L are examples of two respiratory symptoms that are not correlated with either dimension.

Additional file 1: **Figure 3.** Scatterplot of sensitivity and 1-specificity for combinations of symptom variables using ‘status’ as a gold standard. **(A)** A rule based on combinations of at least one of 5 symptoms. The red dot in the left plot identifies the current case definition based on respiratory symptoms (A|Z|C|B|T). The green and blue dots are sets of largely non-respiratory symptoms used for comparison. **(B)** A rule based on one or more of respiratory symptoms (coryza, cough, sore throat or shortness of breath, Z|C|B|T) AND one or more of 5 non-respiratory symptoms. The table shows the estimated values for the example sets highlighted in the figure.

Additional file 1: **Figure 4.** Posterior distributions of the sensitivity (upper row) and specificity (lower row) of both the symptoms (headache OR general weakness OR anosmia OR muscle pain OR joint pain) and the PCR assay, estimated using Bayesian Latent Class Analysis, based on three chains of 20,000 iterations after a burn-in of 5,000.

Additional file 1: **Figure 5.** Scatterplot of sensitivity and 1-specificity for combinations of symptom variables estimated using Bayesian Latent Class Analysis. **(A)** A rule based on combinations of at least one of 5 symptoms. The red dot in the left plot identifies the current case definition based on respiratory symptoms (A|Z|C|S|B). The green and blue dots are sets of largely non-respiratory symptoms used for comparison. **(B)** A rule based on one or more of respiratory symptoms (cough, sore throat, coryza or shortness of breath, Z|C|S |B) AND one or more of 5 non-respiratory symptoms.

Additional file 1: **Figure 6.** Decision tree built using a machine learning algorithm that minimises the misclassification of cases and non-cases. The thickness of the lines denotes the proportion of the population heading down each branch. Darker shading indicates greater node purity
